# Supplementary material for: A New Fluorescent Chemosensor for Cobalt(II) Ions in Living Cells Based on 1,8-Naphthalimide
Source: Molecules. 2019 Aug 26;24(17):3093. doi: 10.3390/molecules24173093 (PMC6749574; doi:10.3390/molecules24173093)
Supplement: Supplementary file 1 [file molecules-24-03093-s001.pdf]

Supporting Information

A New Fluorescent Chemosensor for Cobalt(II) Ions in Living Cells Based on 1,8-Naphthalimide

Yulong Liu [1,†], Liu Yang [1,†] Lu Li [1], Youquan Guo [2], Xiaoxiao Pang [1], Ping Li [1], Fei Ye [1] and Ying Fu [1,*]

1. Department of Applied Chemistry, College of Science, Northeast Agricultural University, Harbin 150030, China
2. Heilongjiang Vocational College of Biology Science and Technology, Harbin 150025, China

* Correspondence: fuying@neau.edu.cn; Tel.: +86-451-5519-0070

† These authors contributed equally to this work.

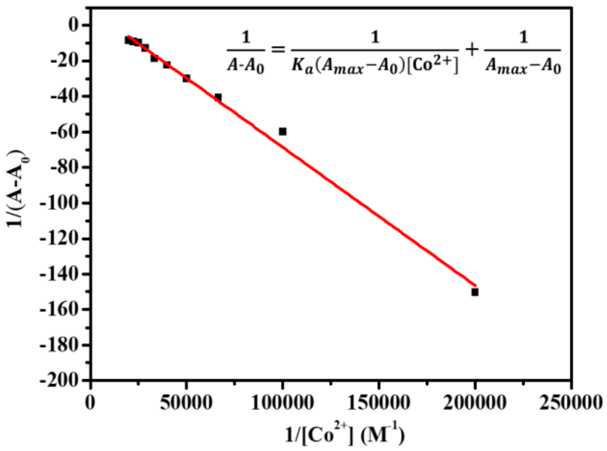

Figure S1. Benesi–Hildebrand plot of sensor **L** in CH_3CN/HEPES (4/1, *v*/*v*, pH=7.4) (1×10^{-5} M) in the presence of Co^{2+}.

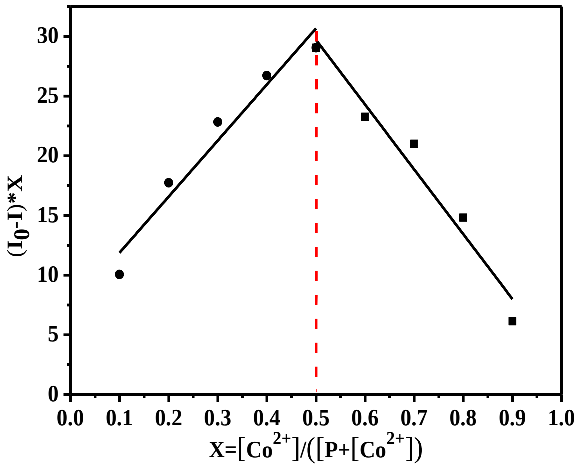

Figure S2. Job's plot analysis of the **L**-Co^{2+} complex in the CH_3CN/HEPES (4/1, *v*/*v*, pH=7.4) solution with the monitoring wavelength set at 450 nm.

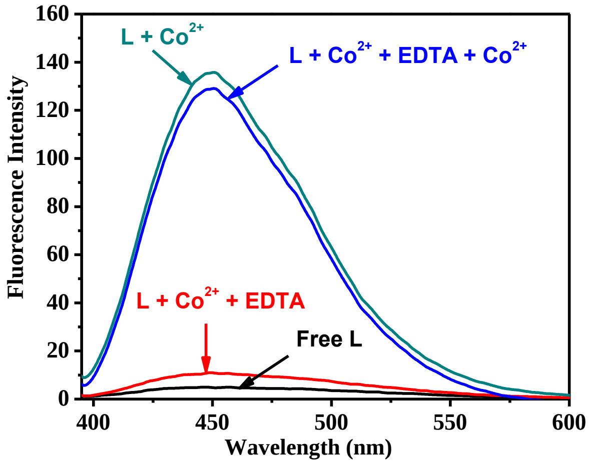

Figure S3. Fluorescence reversibility of **L** upon the detection of Co^{2+}. Fluorescent changes of **L** after the addition of Co^{2+}, EDTA, and Co^{2+}, in that order, in CH_3CN/HEPES (4/1, *v*/*v*, pH=7.4) at 25 ˚C.

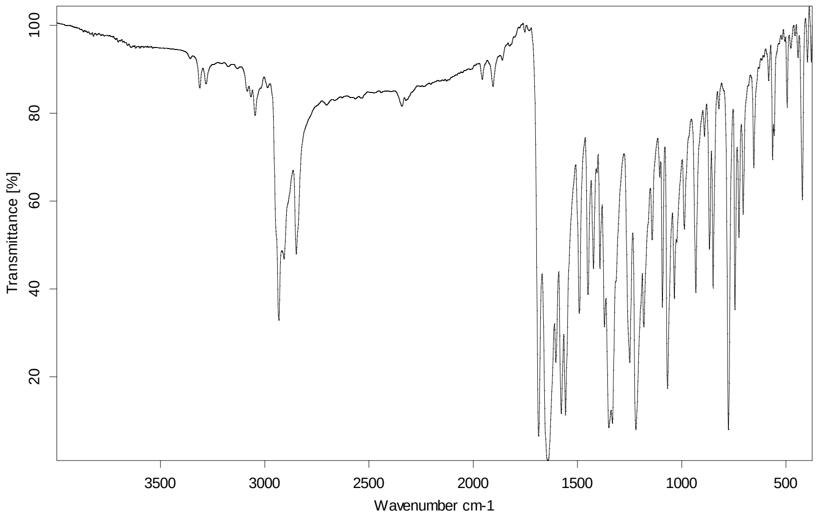

Figure S4. IR spectrum of Compound 1.

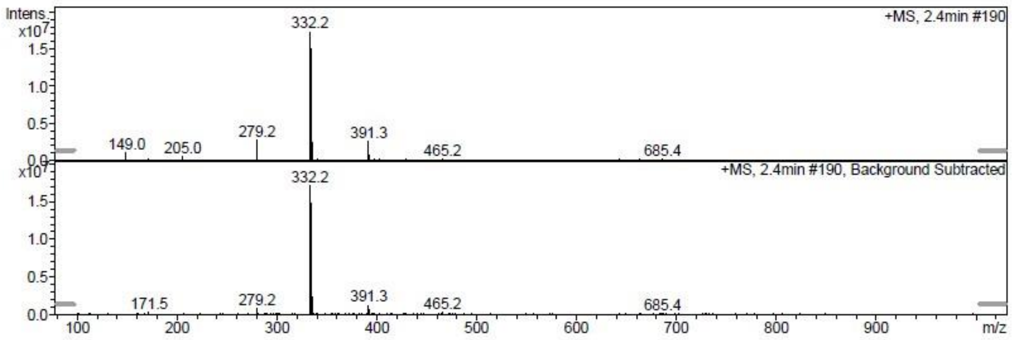

Figure S5. Mass spectrum ($M+H^+$) of Compound 1.

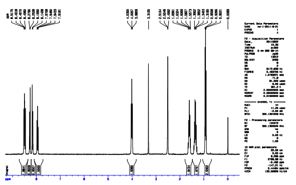

Figure S6. ^{1}H NMR spectrum of Compound 1.

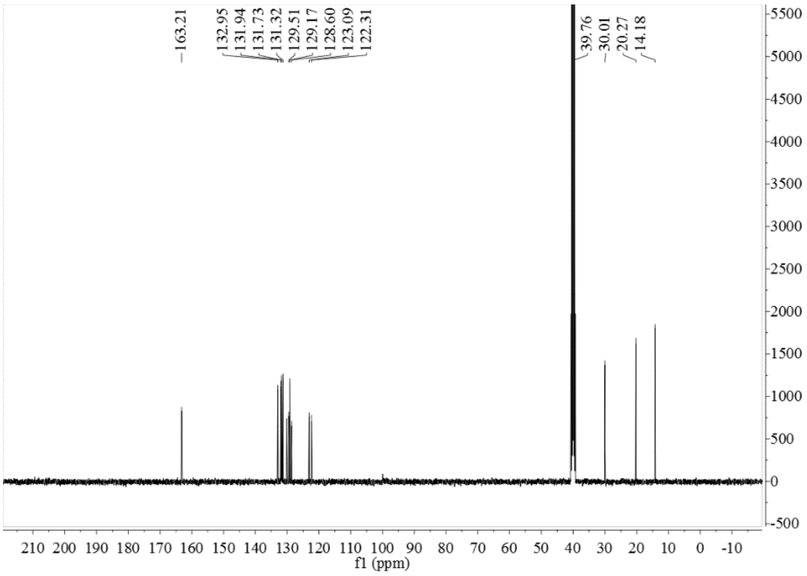

Figure S7. ^{13}C NMR spectrum of Compound 1.

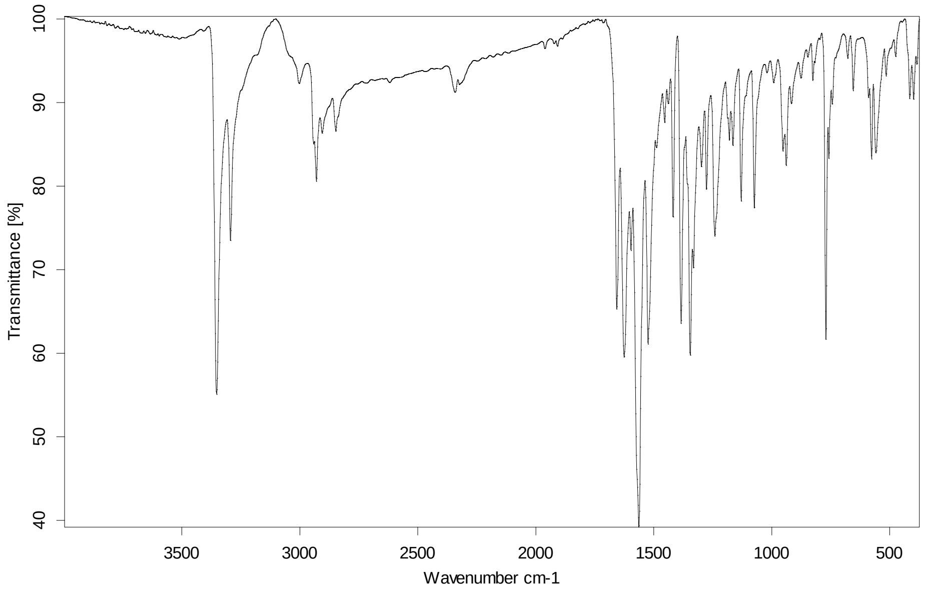

Figure S8. IR spectrum of Compound 2.

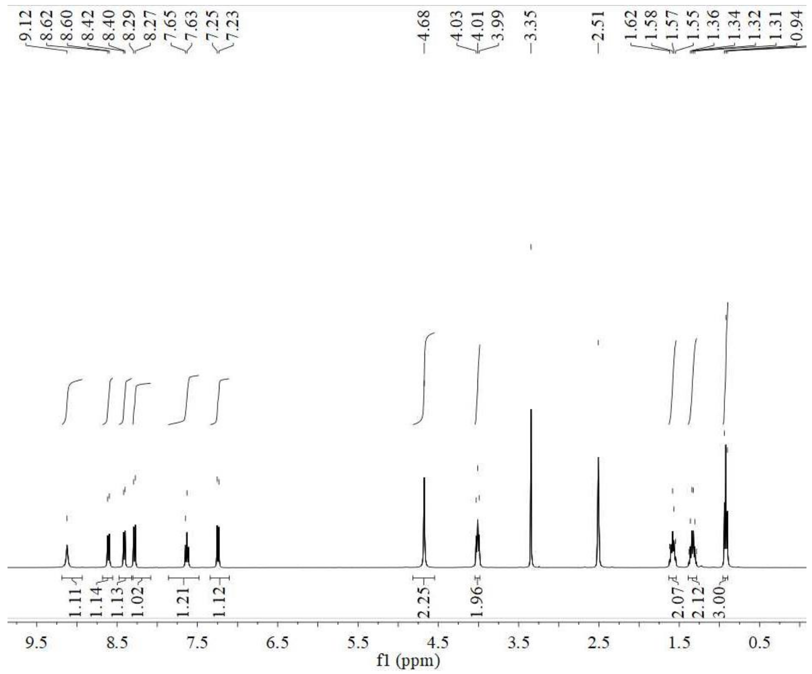

Figure S9. ^{1}H NMR spectrum of Compound 2.

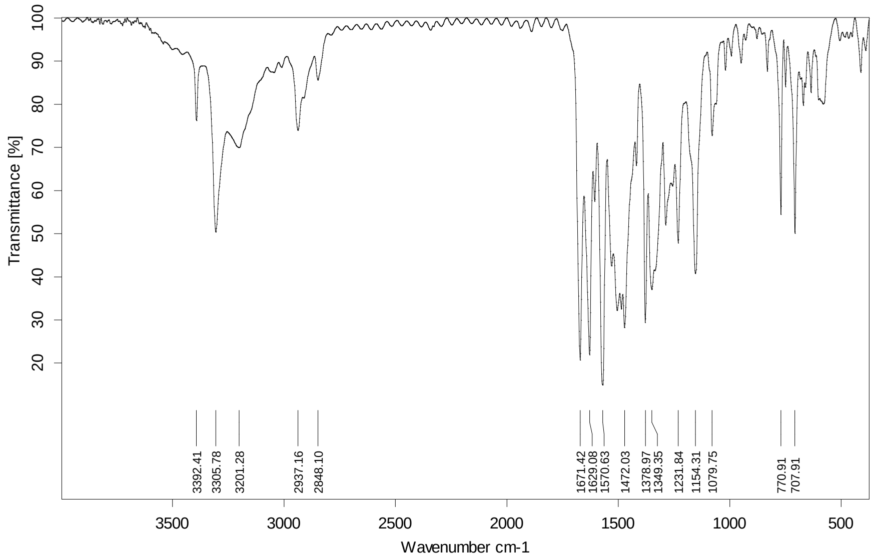

Figure S10. IR spectrum of Compound **L**.

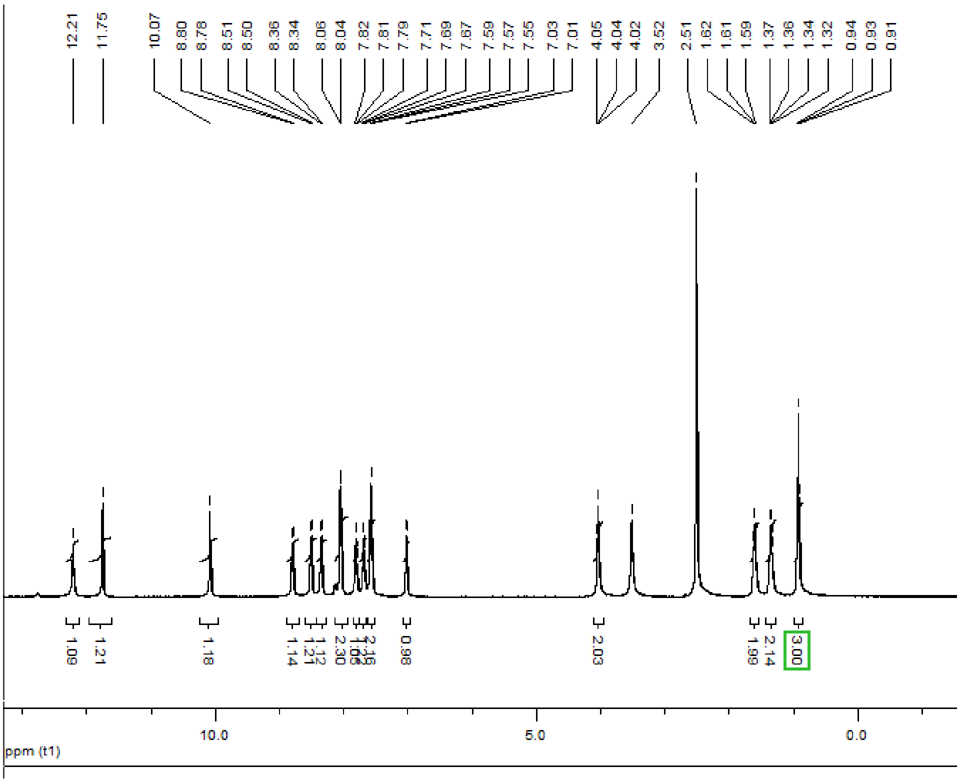

Figure S11. ^{1}H NMR spectrum of Compound **L**.

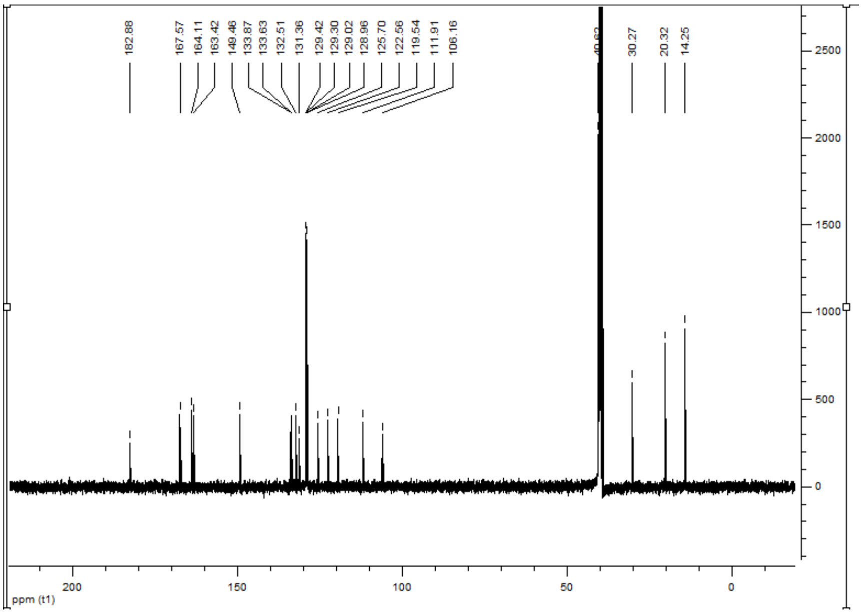

Figure S12. ^{13}C NMR spectrum of Compound **L**.

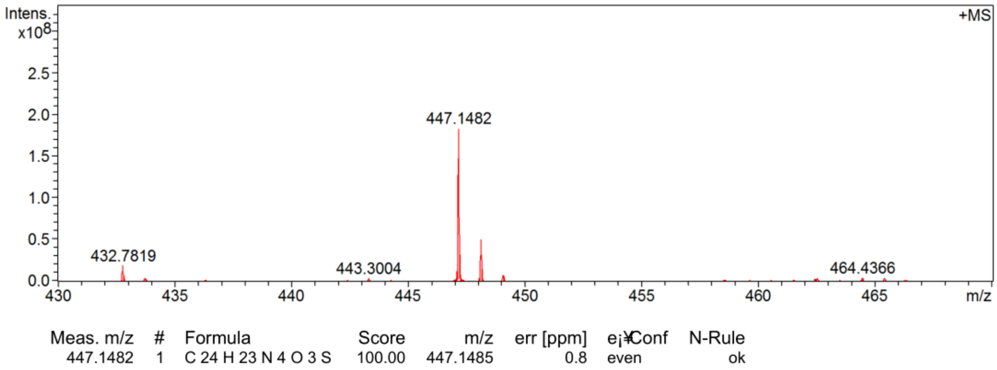

Figure S13. Mass Spectrum ($M+H^+$) of Compound **L**.
